# Nonlinear transcriptomic response to dietary fat intake in the small intestine of C57BL/6J mice

**DOI:** 10.1186/s12864-016-2424-9

**Published:** 2016-02-09

**Authors:** Tenzin Nyima, Michael Müller, Guido J. E. J. Hooiveld, Melissa J. Morine, Marco Scotti

**Affiliations:** The Microsoft Research - University of Trento Centre for Computational and Systems Biology, Rovereto, Italy; Nutrition, Metabolism and Genomics group, Division of Human Nutrition, Wageningen University, Wageningen, The Netherlands; Norwich Medical School, University of East Anglia, Norwich, UK; GEOMAR Helmholtz Centre for Ocean Research, Kiel, Germany

**Keywords:** ABC transporters, Acute-phase response, Cholesterol homeostasis, Cholesterol transport and efflux, Inflammatory response, Small intestine absorption, Transcriptomic response to fat intake

## Abstract

**Background:**

A high caloric diet, in conjunction with low levels of physical activity, promotes obesity. Many studies are available regarding the relation between dietary saturated fats and the etiology of obesity, but most focus on liver, muscle and white adipose tissue. Furthermore, the majority of transcriptomic studies seek to identify linear effects of an external stimulus on gene expression, although such an assumption does not necessarily hold. Our work assesses the dose-dependent effects of dietary fat intake on differential gene expression in the proximal, middle and distal sections of the small intestine in C57BL/6J mice. Gene expression is analyzed in terms of either linear or nonlinear responses to fat intake.

**Results:**

The highest number of differentially expressed genes was observed in the middle section. In all intestine sections, most of the identified processes exhibited a linear response to increasing fat intake. The relative importance of logarithmic and exponential responses was higher in the proximal and distal sections, respectively. Functional enrichment analysis highlighted a constantly linear regulation of acute-phase response along the whole small intestine, with up-regulation of *Serpina1b*. The study of gene expression showed that exponential down-regulation of cholesterol transport in the middle section is coupled with logarithmic up-regulation of cholesterol homeostasis. A shift from linear to exponential response was observed in genes involved in the negative regulation of caspase activity, from middle to distal section (e.g., *Birc5*, up-regulated).

**Conclusions:**

The transcriptomic signature associated with inflammatory processes preserved a linear response in the whole small intestine (e.g., up-regulation of *Serpina1b*). Processes related to cholesterol homeostasis were particularly active in the middle small intestine and only the highest fat intake down-regulated cholesterol transport and efflux (with a key role played by the down-regulation of ATP binding cassette transporters). Characterization of nonlinear patterns of gene expression triggered by different levels of dietary fat is an absolute novelty in intestinal studies. This approach helps identifying which processes are overloaded (i.e., positive, logarithmic regulation) or arrested (i.e., negative, exponential regulation) in response to excessive fat intake, and can shed light on the relationships linking lipid intake to obesity and its associated molecular disturbances.

**Electronic supplementary material:**

The online version of this article (doi:10.1186/s12864-016-2424-9) contains supplementary material, which is available to authorized users.

## Background

Overconsumption of food that are rich in saturated fats leads to excessive energy intake and is strongly linked to metabolic disorders such as obesity, diabetes, cardiovascular diseases and some forms of cancer [1–5]. The intestine and the liver contribute significantly to control whole body and plasma lipid metabolism, being involved in lipid absorption and synthesis [2]. As the primary source of dietary fat uptake, the small intestine plays a key role in governing nutritional health [6–10]. The intestinal absorptive capacity is enhanced by numerous finger-like projections of the mucosal membrane called villi, and there exist regionalized anatomic and physiological differences from proximal duodenum to distal ileum. Food breakdown starts in the mouth and the stomach, but continues in the small intestine. In the proximal section of small intestine, food breakdown occurs with the help of enzymes released from multiple organs (gall bladder, pancreas and Brunner’s gland), and the absorption of phospholipids takes place [2]. The middle section has longer villi in comparison with the proximal and distal sections. It covers nearly half of the intestinal length and is characterized by the highest absorptive capacity. The distal section contains shorter villi and shows less absorptive capacity. Fat-soluble vitamins are preferentially absorbed in different sections of the intestine: vitamin A is mostly absorbed in the proximal intestine, while vitamin D absorption is maximum in the median intestine, and the absorption of vitamin E and K mainly occurs in the distal intestine [11]. Intestinal cholesterol uptake depends on the competing activities of NPC1L1, ABCG5 and ABCG8 present in the apical membrane [12]. CD36 (Cluster of Differentiation 36), an integral membrane protein, participates in the uptake of long chain fatty acids (LCFAs) by intestinal enterocytes and contributes to optimal chylomicron formation in proximal section [13, 14], while CD36-independent absorption processes take place in the distal section [14]. CD36 disappears from the luminal side of intestinal villi early during the postprandial period and operates as a lipid sensor responsible for the dietary activation of intestinal ERK1/2 [15]. The CD36/lipid-dependent modulation of ERK1/2 associates with an increase of two key chylomicron synthesis proteins (i.e., apolipoprotein B48 and microsomal triglyceride transfer protein). During lipid absorption, intestinal enterocytes synthesize apoA-IV that, by increasing the number of triglyceride (TG) rich lipoproteins, enhances TG transport in proximal intestine [16].

**Fig. 1:**
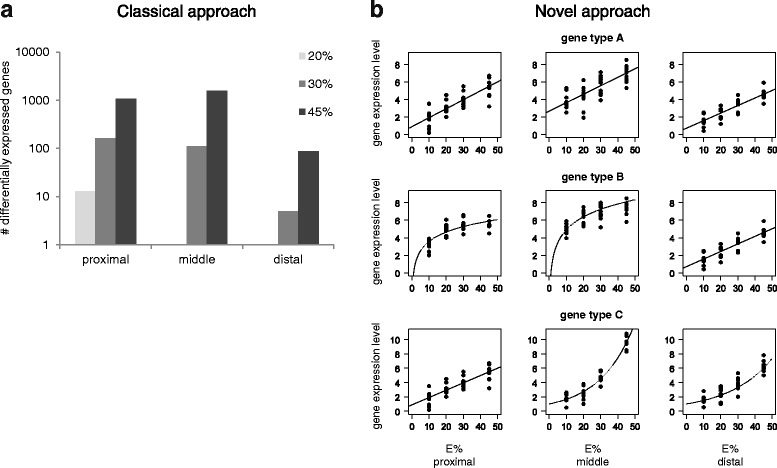
Comparison between classical and novel approach for identifying differentially expressed genes. We use as a reference the case study of mice fed with four levels of fat representing 10, 20, 30 and 45 % of total energy intake [18]; gene expression is measured in three sections of small intestine: **a** In the classical approach, the total number of differentially expressed genes is determined by comparing the control diet (10 E%) with treatments (20, 30 and 45 E%). Gene counts refer to results of de Wit et al. [18]; y-axis on log10 scale. **b** In the novel approach, all expression levels of each gene (in the three sections) are considered to fit linear or nonlinear (i.e., logarithmic and exponential, in this case) response types. Three hypothetical patterns are described: (I) gene type A preserves a linear response in the three sections of small intestine; (II) gene type B displays overload (i.e., logarithmic response) in the proximal and middle sections; (III) gene type C shows exponential responses in most downstream sections (i.e., middle and distal parts of small intestine). Transcriptomic response of gene type A is gene-specific while the patterns observed for gene type B and C are section-specific

The small intestine acts as a gatekeeper between the diet and the body and can directly metabolize or block nutrient uptake. Recent studies have demonstrated a strong intestinal transcriptomic response to dietary fat intake. Kondo et al. found high fat-induced up-regulation of lipid metabolism-related genes (e.g., *Mod1*, *Cyp4a10*, *Acot1* and *Acot2*) in the small intestine of C57BL/6J mice, with negligible effects observed in the liver, muscle and white adipose tissue [6]. Fat intake triggers the down-regulation of ABC half-transporters (*Abcg5* and *Abcg8*) in liver and intestine thus leading to increased levels of sterols in diabetic rats [17]. Biological processes like inflammatory response and cell cycle were found to be highly up-regulated in the small intestine of C57BL/6J mice during dietary fat-induced development of obesity and insulin resistance [7]. The absorption capacity of intestine displays adaptability in response to dietary fat composition such as enhanced intestinal cell proliferation, synchronization of fatty acid uptake and lipoprotein secretion, and altered transport processes [8, 9]. Section-wise studies have focused on gene-specific responses: (I) Simon et al. found increased distal gut hormone response to a high fat diet in apoA-IV knockout mice [16]; (II) Nassir et al. observed sharp decreasing gradient in CD36 levels from proximal to distal intestine [14]. de Wit et al. showed prominent effect of dietary-fat doses on gene expression, mainly in the proximal and middle sections [18]. They concluded that differentially expressed genes correlated with the development of obesity, and the main shift towards an obese phenotype was observed when an amount of energy included between 20 and 30 % was derived from fat. Previous research studies investigated the consequences of fat intake on transcriptomic response of intestine by individual, pairwise comparisons between control (i.e., baseline) diets and treatments (e.g., high fat vs. low fat) [6, 7, 19]. Their goal was detecting a significant difference between control and treatments, rather than quantifying the strength of the response as a function of different levels of fat intake. Our work is a re-analysis of the data produced by de Wit et al. [18]. We propose a novel approach to study whether linear or nonlinear response types characterize gene expression in the small intestine (i.e., dietary fat intake represents the independent variable and gene expression level is the dependent variable). We investigated dose-dependent transcriptomic response to dietary fat along the horizontal axis (proximal, middle and distal sections) of the small intestine of C57BL/6J mice. Mice were fed with 10, 20, 30 or 45 energy% (E%) derived from fat for four weeks (*n* = 10 mice per diet group) and corresponding gene expression levels served to fit dose-dependent responses. Due to the unique morphological and functional characteristics of each intestinal section [2, 9, 19, 20], we expected to observe variations in transcriptomic response from proximal to distal section. Our focus was mainly upon evaluating possible nonlinear relationships between gene expression and dietary fat percentage in the three sections. We aimed at categorizing changes in the response types of differentially expressed genes (i.e., linear response; nonlinear responses: logarithmic, exponential, quadratic or cubic) among each of the three sections of the small intestine (Fig. 1). The main advantage of studying continuous transcriptomic responses as a function of dietary fat intake is related to the chance of combining the information of different treatments into a unique picture. This helps in better understanding how the response to highest levels of fat intake is attained. Thus, we can investigate whether biological processes are overloaded by highest levels of fat content (i.e., logarithmic response type) or modulated in order to cope with them (i.e., linear or exponential response types). The presence of a constant, linear response type along the whole small intestine can indicate spatial-independent mechanisms (i.e., this applies to processes that respond in a gene-specific manner, independently of the intestine section where they occur). For example, such an approach can be used to illustrate whether an obese phenotype (I) corresponds to a ‘tipping point‘ of the system (i.e., exponential response vs. linear and logarithmic responses) or (II) is limited by the metabolic capacity of the system (i.e., logarithmic response vs. linear and exponential responses). Moreover, distinguishing among the shape of the response types in differentially expressed genes adds a further qualitative level to the description of the biological processes (i.e., in addition to the magnitude and direction of the regulation). The broadest goal of our study was investigating whether the response types are gene-specific (i.e., a given gene always exhibits the same response type, regardless of intestinal section) or intestinal section-specific (i.e., for the same gene, the response type displays unique patterns in the three intestine sections).

## Methods

### Ethics statement

The institutional and national guidelines for the care and use of animals were followed. The experiment was approved by the Local Committee for Care and Use of Laboratory Animals at Wageningen University.

### Dietary intervention

At 12 weeks of age, mice were fed for an initial period of four weeks with a control diet containing 10 % of energy intake from fat. The main reason for this relatively long run-in period was that we wanted to be sure that the effects of chow diet were highly diluted/not present when starting the dietary intervention. In addition, since we wanted to investigate the effect of diet-induced obesity in adults, we choose to start the experiment when the mice were 16 weeks old (mice aged 12 weeks are considered young adults that are still growing). After the initial period, mice were divided into four groups that received 10, 20, 30 or 45 % kcal from fat (see Additional file 1, Table A1.1 for dietary composition). The dietary intervention lasted four weeks and then mice were killed by cervical dislocation after five hours fasting and anesthesia with 1.5 % isoflurane. The isoflurane was evaporated in a vaporizer using a mixture consisting of 70 % nitrous oxide and 30 % oxygen. Ten biological replicates were carried out for each diet group in the three sections of the small intestine (i.e., altogether we analyzed 120 samples).

### Microarray data

We analyzed microarray transcriptomic data from the intestinal mucosa of male C57BL/6J mice. The small intestine was divided in three equal parts: proximal, middle and distal section. These three parts were chosen because of practical reasons; when dissecting the small intestine one has to process the tissue quickly to avoid RNA degradation. The 1st part (i.e., proximal section) consists of the duodenum plus proximal jejunum, the 2nd part (i.e., middle section) corresponds to jejunum, and the 3rd part (i.e., distal section) includes the distal jejunum and ileum. Detailed protocols on dietary intervention and RNA extraction are described by de Wit et al. [18]. The microarray platform used for this study is nugomm1a520177mmentrezg, a custom Affymetrix mouse array containing 16,269 probesets. The NuGO arrays are custom Affymetrix GeneChip arrays designed by the European Nutrigenomics Organisation (NuGO) and manufactured by Affymetrix. These arrays contain in part common probe sets that are also present on standard Affymetrix arrays and in part newly designed probe sets (GEO platform GPL7440). The microarray data used for our analyses are MIAME compliant, available at GEO (accession number GSE26300). Data pre-processing and quality assessment, statistical analysis to identify differentially expressed genes and gene set enrichment analysis have been carried out in the R Statistical Environment [21].

### Data pre-processing and quality assessment

We used the affyPLM (PLM = Probe Level Model) library for data preprocessing and quality assessment. We applied the fitPLM function that fits iterative reweighted least squares M-estimation regression to the probe intensity [22]. Background intensities (optical noise and non-specific binding) were adjusted with the GCRMA library [23]. Such adjustment is obtained via estimators derived from a statistical model that uses probe sequence information. The GCRMA library has been shown to perform particularly well in adjusting background intensity in Affymetrix Genechips [24]. After background adjustment, technical variability between arrays was adjusted by quantile normalization [25]. The quality of the PLMset object was assessed by plotting Relative Log Expression (RLE) and Normalized Unscaled Standard Error (NUSE) [26]. Genes with low variability across samples are usually considered as not expressed. This is motivated by the observation that, in general, unexpressed genes are detected most reliably through low variability of their features across samples. Non-specific filtering of the genes was made with the genefilter library [27, 28]. The pOverA R function was used for variance-based filtering; genes with unlogged intensity above five, in at least five arrays, were chosen for the subsequent analysis. Genes without Entrez Gene ID and Affymetrix quality control probe-sets were excluded. After data pre-processing and quality assessment we selected 14,952 genes in the proximal section, 14,933 in the middle and 14,925 in the distal.

### Statistical analysis to identify differentially expressed genes

We investigated the statistical relationship between gene expression and dietary fat intake. We fitted linear and nonlinear (i.e., logarithmic, exponential, quadratic and cubic) responses describing gene expression levels (dependent variable) as a function of dietary fat intake (independent variable). We considered nonlinear responses that reflect: (I) blunted differential expression at higher fat intake, which may indicate overloading of the relevant biological process (logarithmic curve); (II) progressively stronger differential expression with increasing fat intake (exponential curve); (III) parabola-like differential expression (quadratic function); (IV) oscillating trends (cubic function). The array data were log2 transformed (GCRMA normalized data). All responses (i.e., linear and nonlinear) were tested by modelling log2 transformed expression as a function of fat intake (i.e., with fat providing 10, 20, 30 or 45 E%). When comparing the diets of 40 mice (*n* = 10 mice per diet group), there was no significant difference with respect to total food intake in grams per day (see Additional file 1, Table A1.2). However, caloric intake increased with increasing fat percentage (and a linear relationship existed between the % of kcal from fat intake and the actual grams of fat intake - i.e., soybean oil and palm oil). Fat intake (the independent variable) was considered as continuous predictor of log2 transformed gene expression. We used the limma library [29] to perform linear regression on GCRMA normalized data (i.e., log2 transformed gene expression), and tested nonlinear responses by modeling expression as a function of logarithmic-, exponential-, quadratic- and cubic-transformed fat intake. To identify differentially expressed genes we performed multiple testing correction, using Benjamini and Hochberg’s false discovery rate (FDR, with 0.1 significance threshold; see [30]). It should be noticed that many studies regularly adopt an adjusted *p*-value threshold of 0.1 for identifying differentially expressed genes (e.g., [31, 32]). In the case that both linear and nonlinear responses were significant for a given gene, we selected the one with the lowest *p*-value. The robustness of our analysis was then tested by comparing the results obtained with two criteria of model selection (i.e., using *p*-values and Akaike’s information criterion - AIC).

### Gene set enrichment analysis

We investigated whether fat-responsive genes were enriched within distinct biological processes (defined as Gene Ontology Biological Process - GOBP), and tested whether each intestinal section displayed unique transcriptomic response to fat intake. We performed hypergeometric tests to functionally characterize groups of differentially expressed genes. We investigated over-represented GOBP in each section (adjusted *p*-value < 0.1; such threshold is commonly used for gene set enrichment analysis - e.g., [33, 34]) using the library HTSanalyzeR [35]. To understand whether specific response patterns (i.e., linear, logarithmic, exponential, quadratic or cubic) characterized certain biological processes, or changed according to the intestinal sections, we assessed trends of GOBP terms passing from the proximal to the distal region. We studied GOBP terms that preserved the same response pattern in different intestinal sections (e.g., proximal: linear - middle: linear - distal: linear), and analyzed those changing response pattern between the sections (e.g., proximal: linear - middle: logarithmic; middle: logarithmic - distal: linear; middle: linear - distal: exponential). A flowchart of the overall analysis is shown in Additional file 2.

## Results

### Linear and nonlinear gene expression

**Fig. 2:**
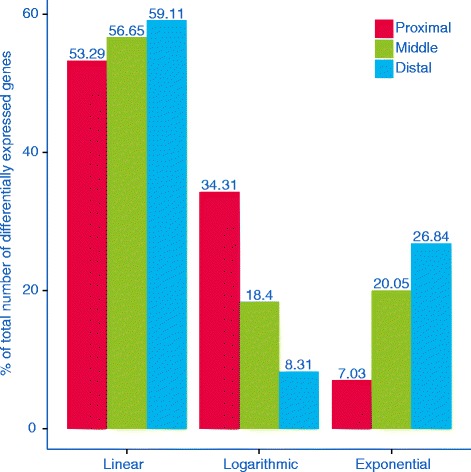
Percentages of differentially expressed genes that responded in a linear, logarithmic and exponential way to fat intake. Histograms are grouped according to response type, while different colors are associated with three small intestine sections. Linear response was highly represented in all sections, while the relative importance of logarithmic (exponential) response decreased (increased) from the proximal to the distal part of small intestine

**Fig. 3:**
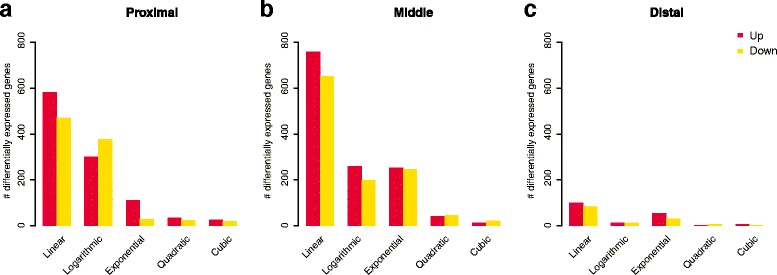
Differentially expressed genes that were either up- or down-regulated in response to increasing fat intake. Results were obtained using the limma library, modeling gene expression as a function of raw fat intake data (i.e., linear model), logarithm-, exponential-, quadratic- or cubic-transformed fat intake values; adjusted *p*-value < 0.1. In each plot, y-axis represents the gene count and x-axis corresponds to response types (linear, logarithmic, exponential, quadratic and cubic). Results refer to three sections of small intestine: **a** proximal; **b** middle; and **c** distal

Pre-processed and filtered data concerning all differentially expressed genes found in the three sections of the small intestine are available in Additional file 3. This file contains details on gene nomenclature (Affymetrix gene ID and gene symbol), response type (lm = linear, log = logarithm, exp = exponential, quad = quadratic, cub = cubic) and direction of change (Up = up-regulated, Down = down-regulated). The count of genes that responded in a significant way (either linear or nonlinear) to fat intake varies between the three sections (Table 1). The highest number of genes showing linear, exponential or quadratic transcriptomic response to fat intake was found in the middle section, but the highest number of genes displaying either logarithmic or cubic patterns of expression in response to fat intake was in the proximal section. The lowest number of differentially expressed genes, for all five response types, was observed in the distal section. The proportion of differentially expressed genes exhibiting a significant linear relationship with the dietary fat intake was always above 53 %. In the proximal and distal sections, the relative importance of logarithmic and exponential response, respectively, was the highest. Both quadratic and cubic response types were of marginal importance in all intestine sections (hereafter, given the fact that quadratic and cubic responses were associated with around 5 % of differentially expressed genes, the focus will be on linear, logarithmic and exponential patterns only). The number of genes displaying a logarithmic response to fat intake dropped from the proximal to the distal section (the percentage decreases from 34.31 % in the proximal to 8.31 % in the distal section). The opposite trend was found for the exponential response: the percentage increases from 7.03 % in the proximal to 26.84 % in the distal section. Thus, although the highest number of genes responding in an exponential way to fat intake was in the middle section, an increasing relative importance of this response was observed from the proximal to the distal part of the small intestine (Fig. 2). In the three sections, if we consider the changes in gene expression that were significantly associated with varying levels of fat intake we observed a prevalence of up-regulated genes (Fig. 3). These results were not affected by the model selection criteria chosen for the analysis (the trends of differentially expressed genes identified using AIC are summarized in the Additional file 4).

**Table 1:** Count of genes that responded to fat intake in a significant, dose-dependent manner

Response (Gene expression vs. Fat intake)	Proximal			Middle			Distal		
	Gene (count)	Relative %	GOBP (count)	Gene (count)	Relative %	GOBP (count)	Gene (count)	Relative %	GOBP (count)
Linear	1053	53.29	25	1410	56.65	27	185	59.11	26
Logarithmic	678	34.31	1	458	18.40	22	26	8.31	-
Exponential	139	7.03	-	499	20.05	9	84	26.84	8
Quadratic	61	3.09	-	87	3.50	7	9	2.88	-
Cubic	45	2.28	-	35	1.41	2	9	2.88	-

Results were extracted using the limma library and are specific to each section of small intestine; adjusted *p*-value < 0.1. Genes are classified based on the mathematical function used to fit their expression in response to fat intake (linear, logarithmic, exponential, quadratic or cubic). For each section, we summarize the relative percentage of genes characterized by the five response types (%) and the number of significant GO terms (Biological Processes - GOBP) found with hypergeometric test (adjusted *p*-value < 0. 1). Only GOBP terms with at least six differentially expressed genes (in the whole small intestine) have been taken into account

### Functional annotation

In the previous section, we classified the genes according to their category of response to fat intake (i.e., using linear, logarithmic, exponential, quadratic and cubic response types). We found two main classes: (I) genes that preserve linear responses along the whole longitudinal axis of small intestine; and (II) genes that alter the shape of their transcriptomic response depending on intestinal section. Due to the progressive absorption that occurs in the small intestine the amount of available fat decreases from the proximal to the distal section. This pattern correlates well with the decrease of the relative importance of overload/logarithmic response, and with the increase in relative importance of exponential response. The broadest objective of our study was to characterize the biological processes that are associated with: (I) genes that maintain linear response types (gene-specific processes), and (II) genes that change their transcriptomic response as a function of fat availability (section-specific processes). To this aim we applied hypergeometric test to investigate the presence of GOBP terms that were significantly enriched with fat-responsive genes. We analyzed lists of genes associated with different response patterns to fat intake (i.e., linear, logarithmic or exponential). First, we considered full lists of genes, including both up- and down-regulated genes. Second, we performed gene set enrichment analysis by taking into account the direction of change (i.e., using either up- or down-regulated genes only). We found terms that were over-represented in more sections and, in some cases, they were characterized by changes in the response type along the longitudinal axis of small intestine (section-specific processes). This served to understand whether GOBP terms in different sections preserved their response type (e.g., APR, acute-phase response exhibited a linear response type in all three sections, including both up- and down-regulated genes, and can be classified as a gene-specific process: thus, fat intake triggered the same response pattern, independently of intestinal sections; Table 2 and Additional files 5 and 6) or showed some changes (e.g., cholesterol homeostasis was characterized by linear response in the proximal section and changed to logarithmic in the middle; mostly up-regulated genes, Table 2 and Additional files 5 and 6). The complete list of genes associated with specific processes (i.e., we do not include details on three generic GOBP terms: metabolic process - GO:0008152; oxidation-reduction process - GO:0055114; transport - GO:0006810) that displayed unique patterns along different sections of the small intestine is summarized in Additional file 6 (details on up- and down-regulated genes are available). Additional files 7, 8 , 9 and 10 list GOBP terms and genes with characteristic response types along the small intestine; they refer to strictly up- and down-regulated processes, respectively. We identified distinctive patterns for inflammation-related pathways (i.e., acute-phase response and negative regulation of caspase activity) and cholesterol-related processes (i.e., cholesterol transport, cholesterol homeostasis and cholesterol efflux).

**Table 2:** Over-represented Gene Ontology Biological Process (GOBP) terms that can be found in various intestinal sections

Section and Responses	GO ID	GOBP	Proximal			Middle			Distal		
			Set Size	Hits	Adjusted *p*-value	Set Size	Hits	Adjusted *p*-value	Set Size	Hits	Adjusted *p*-value
Prox-Mid-Dist (all linear)	GO:0006953	Acute-phase response	22	5	0.09	22	6	0.08	21	3	0.03
Prox-Mid (lm - lm)	GO:0006629	Lipid metabolic process	184	33	<0.01	183	46	<0.01			
	GO:0006631	Fatty acid metabolic process	68	19	<0.01	67	28	<0.01			
	GO:0006635	Fatty acid beta-oxidation	21	9	<0.01	21	11	<0.01			
	GO:0006637	Acyl-CoA metabolic process	19	7	<0.01	19	6	0.04			
	GO:0007040	Lysosome organization	18	5	0.03	18	6	0.04			
	GO:0015031	Protein transport	386	45	0.01	387	60	<0.01			
	GO:0022900	Electron transport chain	69	14	<0.01	69	18	<0.01			
	GO:0055085	Transmembrane transport	390	42	0.07	391	57	0.02			
Prox-Mid (lm - log)	GO:0042632	Cholesterol homeostasis	33	11	<0.01	33	4	0.10			
Prox-Mid (lm - exp)	GO:0030301	Cholesterol transport	15	7	<0.01	15	4	0.03			
Mid-Dist (lm - lm)	GO:0006520	Cellular amino acid metabolic process				16	5	0.07	16	2	0.05
	GO:0016042	Lipid catabolic process				69	14	0.05	69	5	0.03
	GO:0051262	Protein tetramerization				17	5	0.09	17	2	0.05
Mid-Dist (lm - exp)	GO:0043154	Negative regulation of caspase activity				36	10	0.02	36	2	0.06
Mid-Dist (log - lm)	GO:0006644	Phospholipid metabolic process				17	4	0.01	17	2	0.05
	GO:0006749	Glutathione metabolic process				24	4	0.04	24	2	0.08
	GO:0045859	Regulation of protein kinase activity				16	4	0.01	16	2	0.05

We summarize total number of genes corresponding to a given GO term in the microarray gene expression (gene set size, labelled as Set Size), count of genes extracted with our analysis (observed hits, labelled as Hits) and adjusted *p*-values (values have been rounded-up to two decimal points; adjusted *p*-value < 0.1). Intestine sections: Prox = proximal; Mid = middle; Dist = distal. Response types: lm = linear; log logarithm; exp = exponential. GOBP terms showing quadratic and cubic response types have been excluded as they can be found in the middle section only (i.e., there are no spatial trends along the small intestine axis; see Table 1)

(I) *Acute-phase response* (GO:0006953): genes belonging to this GOBP term showed a constantly linear response in all three sections of small intestine, with mixed direction of regulation (i.e., both up- and down-regulated genes; see Additional file 6). The only gene differentially expressed in all three sections was *Serpina1b* (up-regulated).
(II) *Negative regulation of caspase activity* (GO:0043154): caspase activity plays an essential role in apoptosis and inflammation. This is a section-specific process: from middle to distal section we observed a change from linear to exponential expression (Table 2). The genes *Birc5* (up-regulated) and *Igbp1* (down-regulated) were significantly responsive in the middle and the distal sections. *Prdx3* and *Gpx1* were differentially expressed and up-regulated in the middle section only.
(III) *Cholesterol transport* (GO:0030301): the response of differentially expressed genes related to this GOBP term changed from linear to exponential when passing from the proximal to the middle section (Table 2). The gene *Abca1* was down-regulated in both proximal and middle small intestine. *Cd36* showed up-regulation proximally, while all differentially expressed genes that showed an exponential response in the middle section were down-regulated (e.g., *Abcg1* and *Scarb1*).
(IV) *Cholesterol homeostasis* (GO:0042632): up-regulated genes involved in this process exhibited overload in the middle section only (i.e., they were linearly significant proximally and in the distal section, but displayed logarithmic response in the middle; Additional files 7 and 8). Most of the genes involved in cholesterol homeostasis were up-regulated (Additional files 6, 7 and 8). For example, the gene *Pla2g10* was up-regulated in both proximal and middle section, and the same positive regulation was displayed by *Apoa4* when moving from the middle to the distal part of small intestine (Additional file 8). These findings illustrate how excessive fat intake can have detrimental consequences (i.e., logarithmic response with an upper limit to gene expression) in processes involved in the maintenance of the cholesterol steady state in cells, especially in the middle section.
(V) *Cholesterol efflux* (GO:0033344): this GOBP term shares many genes with cholesterol transport and homeostasis, but with a less sharply defined behavior. Up-regulated genes showed logarithmic and linear response in the middle and distal sections, respectively (e.g., *Apoa4*). Down-regulated genes displayed linear and exponential response in proximal and middle parts, respectively (e.g., *Abca1*). The overload of the process that regulates steady state of cholesterol within cells (i.e., cholesterol homeostasis) represents a bottleneck, and the negative, exponential response found for cholesterol transport should be considered in relation to it (with the sharp down-regulation that is triggered by the highest level of fat intake only; i.e., 45 E%). It seems that the directed movement of cholesterol into or between cells is impaired when cholesterol level reaches its carrying capacity.

In summary, by completing the analysis of transcriptomic response patterns (i.e., linear and nonlinear response types) with functional annotation we highlighted three main mechanisms of action in the small intestine: (I) acute-phase response (an inflammatory-related process) is not section-specific and exhibits a linear regulation along the whole small intestine; (II) lipid absorption and transportation are particularly active in the middle section, but the coupling with other overloaded functions can limit the processing capacity (see the logarithmic, up-regulation of cholesterol homeostasis and associated exponential, down-regulation of cholesterol transport); (III) in the distal section, an exponential response characterizes the interplay between up- and down-regulated genes involved in the negative regulation of caspase activity, likely having a role in apoptosis and inflammation.

## Discussion

Excessive saturated fat intake leads to metabolic disorders [1–5]. Especially in the small intestine, studies have shown connections to altered lipid metabolic functions or illustrated its role in the development of obesity and diabetes [6, 7]. However, it is quite rare to find analyses on intestinal section-specific response to dietary fat, except for cases related to intestine resection that discuss the side effect after the loss of a single intestinal section. Differences in the absorption capabilities of intestine sections are caused by changes in gene expression [13, 16]. de Wit and colleagues analyzed the transcriptomic variation between dietary groups (e.g., 20 vs. 10 %, 45 vs. 10 % kilocalories from fat), specific to three sections of small intestine of mice [18]. In their experiment, mice were fed diets that differed in dietary fat and carbohydrate content; the respective diets contained 10, 20, 30 or 45 % kcal fat, which was termed as fat intake (within the field of nutrition it is generally accepted to refer to macronutrient content of the diets as percentage of calories derived from fat). Our goal was to re-analyze their data to assess linear and nonlinear transcriptomic response to fat intake by modeling gene expression as a function of fat intake %. The percentage of calories derived from fat intake represented the independent variable, even though such percentage might have been different from the relative amount of calories from fat that was effectively sensed by enterocytes. Moreover, the absorption capacity of enterocytes could be modulated by dietary fat (but in our study the type of fat that varied was always palm oil; i.e., C16:0). We investigated whether the transcriptomic response type was related to specific intestine sections (thus being potentially triggered by fat availability along the longitudinal axis), or uniquely associated with specific genes and biological processes (and preserved in all intestine sections, without any change along the longitudinal axis). Due to progressive absorption along the intestine, we expected higher amount of fat to be available in the proximal section and a progressive decrease towards the distal section. Furthermore, it can be the case that the quantity of dietary fat exceeds the absorption capacity of the proximal section, and thus “overflows” to the middle and distal sections. Moreover, the middle section has greater absorptive area than proximal and distal sections, and is known to be highly affected by the dietary fat [7]. Our results corroborate previous findings, confirming that the highest number of differentially expressed genes is in the middle section of small intestine (the proximal part has a relevant metabolic role, while the middle and distal sections are mainly dedicated to fat absorption and transportation processes; see Table 1). However, two new and clear patterns emerged from our analysis: (I) most of the genes characterized by a significant relationship between expression levels and dietary fat intake exhibited linear responses (i.e., their activity was not overloaded, even at high fat concentration), and the prevalence of linear relationships was persistent along the whole small intestine; (II) the relevance of nonlinear relationships linking fat intake to gene expression levels was section-specific and reflected the progressive reduction of fat availability (due to intestinal absorption) along the longitudinal axis of the small intestine (i.e., the relative importance of logarithmic responses, which can be associated with overload mechanisms, decreased from proximal to distal section, while the relative importance of exponential responses increased when moving towards the distal part). The relevance of quadratic and cubic response types was marginal both in absolute and relative terms, in all sections. Throughout the small-intestine, we observed biological processes that responded to fat intake in a single section, and other processes with either unaltered (e.g., acute-phase response was linear in all three sections; Table 2) or varying responses across the sections (e.g., cholesterol transport changed from linear response in the proximal section, with both up- and down-regulated genes, to exponential response in the middle section, with down-regulated genes only; Additional file 6).

When analyzing differentially expressed genes, we observed that over-representation of APR was linked to fat intake through a linear relationship in all three intestinal sections (Table 2). Increasing dietary fat intake caused intestinal inflammatory transcriptomic response and led to linear increase in APR. This response did not show a univocal pattern and was associated with both up- and down-regulated genes (e.g., *Serpina1b* was up-regulated; *Reg3b* and *Reg3g* were down-regulated). Serine protease inhibitor (*Serpina1b*) was found to be differentially expressed and up-regulated in all three sections. Some studies have shown that the presence of lipopolysaccharide (LPS) in the small intestine stimulates pro-inflammatory mediators that are activators of insulin resistance [36, 37]. The resultant inflammatory reaction causes APR and its prolonged activation is seen with increased plasma levels of small, low-density lipoproteins (LDLs) [38, 39]. Moreover, during inflammation plasma triglyceride and very low-density lipoprotein (VLDL) levels rise, while high-density lipoprotein (HDL) level declines [38]. In the present study, *Reg3g* and *Reg3b* were down-regulated both in proximal and middle small intestine. The protein encoded by these genes (REG3G and REG3B) belong to the family of C-type lectins and are secreted by epithelial cells and Paneth cells. Their expression is reduced in the small intestine of mice fed alcohol compared with control mice, and the effect is more pronounced in the proximal part [40]. REG3G and REG3B play a protective role against gram-positive and gram-negative bacteria, respectively [41, 42]. REG3G is essential for maintaining a zone that physically separates the lumina bacteria from the small intestinal epithelial surface [43]. Dysregulation of a microorganism-induced program of epithelial cell homeostasis and repair can result in chronic inflammatory responses, and is associated with the development of colon cancer [38, 42]. Down-regulation of *Reg3g* and *Reg3b* is likely to result in increased bacterial colonization of the intestinal epithelial surface, with consequent induction of inflammation. The oncogenic transcription factor *Stat3* was down-regulated in both the proximal and the middle section, and the same pattern holds in middle and distal sections for *Saa2*, a member of the family of APR proteins that is usually up-regulated during infection, tissue damage or inflammation disease [44].

A complex scenario characterizes cholesterol, with a prevalence of up- and down-regulation for what concerns cholesterol homeostasis and transport, respectively (Additional files 5, 6, 7, 8, 9 and 10). In the middle section, cholesterol homeostasis was overloaded (i.e., logarithmic response), while cholesterol transport was particularly impaired in presence of highest levels of fat intake (i.e., exponential response). In the middle section, we observed the up-regulated, logarithmic response in the expression of cholesterol homeostasis genes (i.e., *Apoa4*, *Cav1* and *Pla2g10*), except for *Cyp7a1* (down-regulated, logarithmic). In different parts of the small intestine, we found down-regulation of ABC transporters: *Abca1* in proximal as well as in the middle section, *Abcg1* in middle section, and *Abcg5* and *Abcg8* in the proximal section (Additional file 6). These genes displayed fat-responsive expression pattern and are involved in cholesterol transport and cholesterol efflux. They were characterized by a change in the response pattern from proximal (linear response) to middle (exponential response) section. These data suggest that high fat concentrations available in the proximal small intestine down-regulate ABC transporters involved in cholesterol efflux (*Abca1*, *Abcg5* and *Abcg8*; Additional files 6, 8 and 10). The linear response of these down-regulated genes stands for relevant effects also in presence of low fat intakes. The negative exponential response observed in the middle section for *Abca1* and *Abcg1* illustrates how only the highest level of fat intake results in the sharp impairment of cholesterol absorption. This is in line with the fact that the middle section is characterized by a higher uptake capacity than the proximal section. Sections that are downstream the proximal small intestine (which consists of the duodenum plus the proximal jejunum) are exposed to smaller amounts of fat and less pronounced effects (except for the case of highest fat content in the diet; i.e., 45 E%). The highest fat concentration was the only one that triggered a strong change, leading to the down-regulation of two ABC transporters in the middle section.

The negative regulation of caspase activity showed a transition from linear response in the middle section to exponential response in the distal part. This process did not present a univocal response pattern and included both up- and down-regulated genes. In the middle and distal sections, there was up-regulation of the gene *Birc5* (baculoviral IAP repeat-containing 5, also known as survivin). *Birc5* is bifunctional, plays a key role in inhibition of apoptosis and regulation of mitosis, and is essential for cell division [45–47]. *Birc5* is mostly linked with carcinogenesis; during early atherogenesis, it shows elevated expression in inflamed macrophage-rich areas [47]. Linear and positive transcriptomic response of genes *Prdx3* (that encodes the protein thioredoxin-dependent peroxide reductase, mitochondrial) and *Gpx1* (that encodes the enzyme glutathione peroxidase 1) was confined to the middle small intestine. Bellafante et al. have shown that PGC-1β overexpression in enterocytes enhances antioxidant enzymes such as *Sod2*, *Gpx4*, *Prdx3*, *Prdx5*, *Txn2* and *Sirt3* [48]. Up-regulation of these enzymes has an antiapoptotic role both in normal mucosa and protumorigenic conditions, and causes a greater increase in the length of the villi of the small intestine. Chu et al. have observed that the targeted disruption of *Gpx1* and *Gpx2* harms two glutathione peroxidase (GPX) isoenzymes [49]. GPX isoenzymes reduce hydroperoxidases in intestinal epithelium and their impairment increases the sensitivity of ileum and colon to bacteria-associated inflammation and cancer. Our results showed linear, up-regulation of *Prdx3* and *Gpx1* in the middle section of small intestine; this can activate antioxidant processes that are relevant for bacteria-associated inflammation, cancer-promoting conditions and tumor progression. Our result suggests that these biological processes were not overloaded in the middle and distal sections, possibly due to the absorption that contributes, in a progressive way, to reduce the amount of fat in the small intestine lumen. The linear, positive responses found in the middle part illustrate the relevance of antiapoptotic processes, even in presence of low fat intake. The exponential, positive response identified in the distal section (specific to *Birc5*) is descriptive of an outstanding activation, only for most extreme values of fat intake.

Our study aimed at identifying changes in differentially expressed genes in three small intestine sections as a response to dietary fat intake. Kondo et al. analyzed differential gene expression among multiple metabolic organs, including small intestine [6]. They showed that some lipid metabolism-related genes (i.e., *Mod1*, *Cyp4a10*, *Hmgcs2*, *Acot1*, *Acot2*, *Pdk4*, *Acaa1b*, *Cpt1*, *Fabp1*, and *Acadl*) were significantly up-regulated in the intestine of both A/J (obesity-resistant) and C57BL/6J (obesity-prone) mice fed with high fat diet. They observed that in the liver of A/J mice, high fat feeding significantly decreased the expression of *Mod1* and *Cyp4a10*. Also, the expression of *Mod1*, *Hmgcs2*, *Acot2* and *Pdk4* was not increased during high fat feeding in the muscle and white adipose tissue. We found that: (I) *Fabp1* was up-regulated and linearly responsive in all the three sections of the small intestine; (II) *Hmgcs2*, *Acot1*, *Acaa1b* and *Acadl* were linearly responsive and up-regulated in the proximal and middle sections; (III) in the proximal and middle sections, the expression of *Pdk4* was up-regulated and displayed a logarithmic response; (IV) *Acot2* was up-regulated and linearly responsive in the proximal section only. Al-Dwairi et al. reported that during diet-induced obesity, ME1 over-expression in small intestine promoted the expression of hepatic genes associated with lipogenesis, cholesterol synthesis and cholesterol uptake [50]. This suggests gatekeeper functionality of the small intestine, with changes in the expression of ME1 that influence metabolic processes in the liver. In our study, the gene *Me1* was up-regulated and logarithmically responsive in the proximal section, while the up-regulation was associated with linear response in the middle and distal sections. Moreover, we found that processes related to cholesterol homeostasis were particularly active in the middle small intestine and only highest fat intake impaired cholesterol transport and efflux (with a key role played by the down-regulation of ATP binding cassette transporters). In the small intestine, de Wit et al. reported that high fat modulates the expression of secreted proteins such as Il18, Fgf15, Mif, Igfbp3 and Angptl4 [7]. They suggested that these signaling molecules might have metabolic effects in liver, muscle and adipose tissue that underlie the development of the metabolic syndrome. In our study, the gene expression of *Il18* was down-regulated (changing from logarithmic response in the proximal to linear response in the middle section), while *Igfbp3* was linearly up-regulated in the middle section only.

## Conclusions

Our approach to modelling nonlinear transcriptomic signatures in the small intestine revealed that - for a range of biological processes including cholesterol transport and homeostasis - there exists a ‘tipping point’ for fat intake, beyond which the relationship between fat intake and gene expression either weakens (logarithmic curves) or strengthens (exponential curves). For example, the shift from linear to exponential response observed for down-regulated ABC transporters (from the proximal to the middle section; Table 3) is representative of the intense absorption capability of the middle section (i.e., only extreme levels of fat intake down-regulate ABC transporters, while no effect is detected for fat intake ≤ 30 E%). Our results highlight the relevance of nonlinear analysis for modelling more precisely the effects of diet on molecular activity in the small intestine. The observation of increasing relative importance of exponential responses and decreasing relative importance of logarithmic responses from the proximal to distal section is in agreement with the hypothesis that when the absorptive capacity of the intestinal epithelia is overloaded, the remaining fat will overflow to more distal sections (i.e., section-specific behavior). Although most of the genes related to inflammatory processes preserved their linear expression pattern along the whole small intestine (i.e., gene-specific behavior; e.g., see *Serpina1b* and *Cd36*), fat intake regulated in a section-specific fashion the transcriptomic response of *Birc5* (Table 3). The regionalized behavior of *Birc5* suggests that antiapoptotic mechanisms are particularly relevant in the middle section of small intestine and can represent an adaptation to counteract the lipotoxic effect of high fat diets on intestinal cells [2, 51]. Future studies should assess whether the nonlinear patterns observed here are influenced by other factors (e.g., microbiome composition), and investigate the relevance of nonlinear responses in other tissues and clinically relevant markers.

**Table 3:**
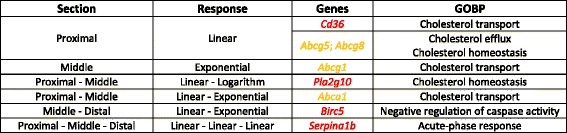
Fat-responsive differentially expressed genes and associated Gene Ontology Biological Process (GOBP) terms

Red colored genes: up-regulated; yellow colored genes: down-regulated. For each gene, site of expression (i.e., Section) and response type/trend (i.e., Response) are shown. These eight genes are the most representative for acute-phase response (in all three intestine sections), cholesterol-related processes (i.e., cholesterol homeostasis, transport and efflux: in proximal and middle section) and negative regulation of caspase (in middle and distal section)

### Availability of supporting data

All supporting data are included as Additional files.

## Additional files

Additional file 1:
**Table A1.1.** Diet composition. Main ingredients are in bold (e.g., palm oil is the main source of saturated fats whereas corn starch is the main source of carbohydrates).**Table A1.2.** Food and caloric intake in week 4. Data refer to the week the mice were killed. We summarized the mean and standard error of the mean (SEM), based on *n* = 10 per diet group. The caloric intake consistently increased from 10 to 45 % diets, but the absolute amounts of food intake varied in a non-significant way. (PDF 376 kb) Additional file 2:
**Schematic representation of the workflow adopted for the analysis of microarray data.** We identified linear and nonlinear response patterns for significantly over-represented GOBP terms. Trends for the response patterns specific to each term were examined from proximal to distal section. (PDF 318 kb) Additional file 3:
**Differentially expressed genes (normalized and processed data) in the three sections of small intestine.** There are 1,976 genes in the proximal section, 2,489 genes in the middle part and 313 genes in the distal section. For each gene we indicated Affymetrix ID, gene symbol, response type (lm = linear, log = logarithmic, exp = exponential, quad = quadratic and cub = cubic), and response direction (either up- or down-regulated). Last 40 columns refer to log2 transformed expression values in the small intestine of mice fed with 10, 20, 30 and 45 E% from fat. (XLSX 2795 kb) Additional file 4:
**Count of genes that responded in a significant, dose-dependent way to fat intake (model selection based on AIC).** We identified the significant responses in three sections of small intestine (limma library, adjusted *p*-value < 0.1). Differential gene expression was modelled as either a linear or nonlinear (i.e., logarithmic, exponential, quadratic or cubic) function of dietary fat intake. For each section, we reported the percentage of genes per response type (%) and the number of significant GO terms (Biological Processes - GOBP; hypergeometric test, adjusted *p*-value < 0. 1). Only GOBP terms with, at least, six differentially expressed genes (in the whole small intestine) have been taken into account. Outcomes obtained by using AIC for model selection were coherent with the trends reported in the manuscript (i.e., model selection based on the lowest *p*-value; see Table 1). In particular, with AIC we found: (I) the prevalence of linear-responding genes in all intestine sections; (II) the decreasing relative importance of the logarithmic response when moving from the proximal to the distal section (while the opposite pattern holds for the exponential response); (III) the marginal relevance of other response types (i.e., quadratic and cubic functions). (PDF 192 kb) Additional file 5:
**Over-represented Gene Ontology Biological Process (GOBP) terms associated with differentially expressed genes in the three intestinal sections.** Genes are classified as linear, logarithmic or exponential, according to the best (i.e., with the smallest adjusted *p*-value) response type describing their expression pattern (as a function of fat intake). For each significant GOBP term (described by Gene set name and Gene set term) we summarize number of genes corresponding to the process (Set size), number of genes found with our analysis (Observed hits) and adjusted *p*-value. Universe size: proximal = 14,952; middle = 14,933; distal = 14,925. (PDF 258 kb) Additional file 6:
**Over-represented Gene Ontology Biological Process (GOBP) terms that can be found in various intestinal sections.** For each GOBP term we specify up- and down-regulated genes (adjusted *p*-value < 0.1; however, most of the results of gene set enrichment analysis are well below this threshold - see Additional file 5). (PDF 296 kb) Additional file 7:
**Over-represented Gene Ontology Biological Process (GOBP) terms that are completely up-regulated.** For each GOBP term we summarize: the total number of genes in the microarray (gene set size, labelled as Set size), the count of genes extracted with our analysis (observed hits, labelled as Hits), and the adjusted *p*-values. Intestine sections: Prox = proximal; Mid = middle; Dist = distal. Response types: lm = linear; log = logarithm; exp = exponential. (PDF 204 kb) Additional file 8:
**Over-represented Gene Ontology Biological Process (GOBP) terms that include up-regulated genes only and can be found in various intestinal sections.** Such GOBP terms can be found in various intestinal sections; lists of (up-regulated) differentially expressed genes associated with each GOBP term are summarized (adjusted *p*-value < 0.1; see Additional file 7). (PDF 286 kb) Additional file 9:
**Over-represented Gene Ontology Biological Process (GOBP) terms that are completelydown-regulated.** Data refer to GOBP terms that can be found in various intestinal sections. The following details are provided for each GOBP term: total number of genes in the microarray (gene set size, labelled as Set size), count of genes extracted with our analysis (observed hits, labelled as Hits), and adjusted *p*-values. Intestine sections: Prox = proximal; Mid = middle; Dist = distal. Response types: lm = linear; log = logarithm; exp = exponential. (PDF 204 kb) Additional file 10:
**Over-represented Gene Ontology Biological Process (GOBP) terms that include down-regulated genes only and can be found in various intestinal sections.** For each term we specify the associated differentially expressed genes (adjusted *p*-value < 0.1; see Additional file 9). (PDF 281 kb)

## Abbreviations

APR: acute-phase response
BP: biological process
FDR: false discovery rate
GO: gene ontology
GOBP: gene ontology biological process
HDL: high-density lipoprotein
LCFA: long chain fatty acid
LDL: low-density lipoprotein
LPS: lipopolysaccharide
NUSE: normalized unscaled standard error
PLM: probe level model
RLE: relative log expression
TG: triglyceride
VLDL: very low-density lipoprotein
