# Supplementary material for: Nonlinear transcriptomic response to dietary fat intake in the small intestine of C57BL/6J mice
Source: BMC Genomics. 2016 Feb 9;17:106. doi: 10.1186/s12864-016-2424-9 (PMC4748552; doi:10.1186/s12864-016-2424-9)
Supplement: Additional file 1: — Table A1.1. Diet composition. Main ingredients are in bold (e.g., palm oil is the main source of saturated fats whereas corn starch is the main source of carbohydrates).Table A1.2. Food and caloric intake in week 4. Data refer to the week the mice were killed. We summarized the mean and standard error of the mean (SEM), based on n = 10 per diet group. The caloric intake consistently increased from 10 to 45 % diets, but the absolute amounts of food intake varied in a non-significant way. (PDF 376 kb) [file 12864_2016_2424_MOESM1_ESM.pdf]

# Additional file 1

| | **10E% fat** | | **20E% fat** | | **30E% fat** | | **45E% fat** | |
|---|---|---|---|---|---|---|---|---|
| | **gm%** | ***kcal%*** | **gm%** | ***kcal%*** | **gm%** | ***kcal%*** | **gm%** | ***kcal%*** |
| Protein | 19 | *20* | 20 | *20* | 22 | *20* | 24 | *20* |
| Carbohydrate | 67 | *69* | 60 | *60* | 53 | *50* | 40 | *35* |
| Fat | 4 | *10* | 9 | *20* | 14 | *30* | 24 | *45* |
| Other | 10 | *1* | 11 | *1* | 11 | *1* | 12 | *1* |
| Total | 100 | *100* | 100 | *100* | 100 | *100* | 100 | *100* |
| kcal/gm | 3.8 | | 4.1 | | 4.3 | | 4.7 | |
| | | | | | | | | |
| **Ingredient** | **gm** | ***kcal*** | **gm** | ***kcal*** | **gm** | ***kcal*** | **gm** | ***kcal*** |
| Casein, lactic | 200 | *800* | 200 | *800* | 200 | *800* | 200 | *800* |
| L-Cystine | 3 | *12* | 3 | *12* | 3 | *12* | 3 | *12* |
| | | | | | | | | |
| **Corn Starch** | **427.2** | *1709* | **325.8** | *1303* | **224.3** | *897* | 72.8 | *291* |
| **Maltodextrin** | **100** | *400* | **100** | *400* | **100** | *400* | 100 | *400* |
| **Sucrose** | **172.8** | *691* | **172.8** | *691* | **172.8** | *691* | 172.8 | *691* |
| | | | | | | | | |
| Cellulose, BW200 | 50 | *0* | 50 | *0* | 50 | *0* | 50 | *0* |
| | | | | | | | | |
| Soybean Oil | 25 | *225* | 25 | *225* | 25 | *225* | 25 | *225* |
| **Palm oil** | **20** | *180* | **65.2** | *586* | **110.2** | *992* | **177.5** | *1598* |
| | | | | | | | | |
| Mineral Mix S10026* | 10 | *0* | 10 | *0* | 10 | *0* | 10 | *0* |
| DiCalcium Phosphate | 13 | *0* | 13 | *0* | 13 | *0* | 13 | *0* |
| Calcium Carbonate | 5.5 | *0* | 5.5 | *0* | 5.5 | *0* | 5.5 | *0* |
| Potassium Citrate, 1 H2O | 16.5 | *0* | 16.5 | *0* | 16.5 | *0* | 16.5 | *0* |
| | | | | | | | | |
| Vitamin Mix V10001* | 10 | *40* | 10 | *40* | 10 | *40* | 10 | *40* |
| Choline Bitartrate | 2 | *0* | 2 | *0* | 2 | *0* | 2 | *0* |
| | | | | | | | | |
| **Total** | **1055** | ***4057*** | **999** | ***4057*** | **942** | ***4057*** | **858** | ***4057*** |

**Table A1.1: Diet composition.** Main ingredients are in bold (e.g., palm oil is the main source of saturated fats whereas corn starch is the main source of carbohydrates).

| Feed intake in grams per day (mean and SEM, n = 10) | | | | | | | |
|---|---|---|---|---|---|---|---|
| 10E% fat | | 20E% fat | | 30E% fat | | 45E% fat | |
| MEAN | SEM | MEAN | SEM | MEAN | SEM | MEAN | SEM |
| 3.396 | 0.060 | 3.279 | 0.072 | 3.404 | 0.034 | 3.196 | 0.073 |
| | | | | | | | |
| Caloric content feed (kcal/gm) | | | | | | | |
| 3.8 | | 4.1 | | 4.3 | | 4.7 | |
| | | | | | | | |
| Caloric intake (kcal per mouse per day); mean (n = 10) | | | | | | | |
| 12.905 | | 13.443 | | 14.635 | | 15.020 | |

**Table A1.2: Food and caloric intake in week 4.** Data refer to the week the mice were killed. We summarized the mean and standard error of the mean (SEM), based on n = 10 per diet group. The caloric intake consistently increased from 10 to 45% diets, but the absolute amounts of food intake varied in a non-significant way.
