# Supplementary material for: Nonlinear transcriptomic response to dietary fat intake in the small intestine of C57BL/6J mice
Source: BMC Genomics. 2016 Feb 9;17:106. doi: 10.1186/s12864-016-2424-9 (PMC4748552; doi:10.1186/s12864-016-2424-9)
Supplement: Additional file 2: — Schematic representation of the workflow adopted for the analysis of microarray data. We identified linear and nonlinear response patterns for significantly over-represented GOBP terms. Trends for the response patterns specific to each term were examined from proximal to distal section. (PDF 318 kb) [file 12864_2016_2424_MOESM2_ESM.pdf]

Raw Data (.CEL)

↓

Data pre-processing (GCRMA, Quantile)
& Quality assessment (RLE & NUSE)

↓

Analysis to identify Differentially Expressed Genes (DEG)

Linear, logarithmic, exponential, quadratic and cubic responses to dietary fat intake (specific to each intestine section; limma library)

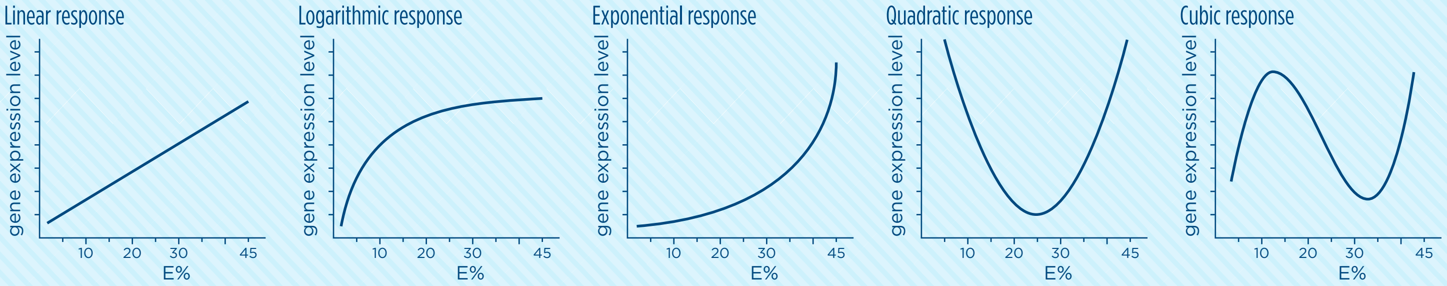

↓

Hypergeometric test to find over-represented Gene Ontology (GO) terms, Biological Processes (BP)

Significant GO terms associated to linear, logarithmic, exponential, quadratic and cubic responses
(specific to each intestine section; HTSanalyzeR library)

↓

Analysis of GOBP terms exhibiting unique trends from proximal to distal small intestine
