# Supplementary material for: Nonlinear transcriptomic response to dietary fat intake in the small intestine of C57BL/6J mice
Source: BMC Genomics. 2016 Feb 9;17:106. doi: 10.1186/s12864-016-2424-9 (PMC4748552; doi:10.1186/s12864-016-2424-9)
Supplement: Additional file 4: — Count of genes that responded in a significant, dose-dependent way to fat intake (model selection based on AIC). We identified the significant responses in three sections of small intestine (limma library, adjusted p-value < 0.1). Differential gene expression was modelled as either a linear or nonlinear (i.e., logarithmic, exponential, quadratic or cubic) function of dietary fat intake. For each section, we reported the percentage of genes per response type (%) and the number of significant GO terms (Biological Processes - GOBP; hypergeometric test, adjusted p-value < 0. 1). Only GOBP terms with, at least, six differentially expressed genes (in the whole small intestine) have been taken into account. Outcomes obtained by using AIC for model selection were coherent with the trends reported in the manuscript (i.e., model selection based on the lowest p-value; see Table 1). In particular, with AIC we found: (I) the prevalence of linear-responding genes in all intestine sections; (II) the decreasing relative importance of the logarithmic response when moving from the proximal to the distal section (while the opposite pattern holds for the exponential response); (III) the marginal relevance of other response types (i.e., quadratic and cubic functions). (PDF 192 kb) [file 12864_2016_2424_MOESM4_ESM.pdf]

Additional file 4

Response (Gene expression vs. Fat intake)	Proximal			Middle			Distal		
	Gene (count)	Relative %	GOBP (count)	Gene (count)	Relative %	GOBP (count)	Gene (count)	Relative %	GOBP (count)
Linear	**1219**	**48.18**	6	**1439**	**50.03**	16	**366**	**50.76**	3
Logarithmic	**719**	**28.42**	7	**443**	**15.40**	11	**45**	**6.24**	-
Exponential	**148**	**5.85**	2	**461**	**16.03**	17	**121**	**16.78**	6
Quadratic	**212**	**8.38**	-	**326**	**11.34**	-	**85**	**11.79**	-
Cubic	**232**	**9.17**	1	**207**	**7.20**	-	**104**	**14.42**	4

Table A4: Count of genes that responded in a significant, dose-dependent way to fat intake (model selection based on AIC). We identified the significant responses in three sections of small intestine (limma library, adjusted p-value < 0.1). Differential gene expression was modelled as either a linear or nonlinear (i.e., logarithmic, exponential, quadratic or cubic) function of dietary fat intake. For each section, we reported the percentage of genes per response type (%) and the number of significant GO terms (Biological Processes - GOBP; hypergeometric test, adjusted p-value < 0. 1). Only GOBP terms with, at least, six differentially expressed genes (in the whole small intestine) have been taken into account. Outcomes obtained by using AIC for model selection were coherent with the trends reported in the manuscript (i.e., model selection based on the lowest p-value; see Table 1). In particular, with AIC we found: (I) the prevalence of linear-responding genes in all intestine sections; (II) the decreasing relative importance of the logarithmic response when moving from the proximal to the distal section (while the opposite pattern holds for the exponential response); (III) the marginal relevance of other response types (i.e., quadratic and cubic functions).
