# Supplementary material for: Nonlinear transcriptomic response to dietary fat intake in the small intestine of C57BL/6J mice
Source: BMC Genomics. 2016 Feb 9;17:106. doi: 10.1186/s12864-016-2424-9 (PMC4748552; doi:10.1186/s12864-016-2424-9)
Supplement: Additional file 5: — Over-represented Gene Ontology Biological Process (GOBP) terms associated with differentially expressed genes in the three intestinal sections. Genes are classified as linear, logarithmic or exponential, according to the best (i.e., with the smallest adjusted p-value) response type describing their expression pattern (as a function of fat intake). For each significant GOBP term (described by Gene set name and Gene set term) we summarize number of genes corresponding to the process (Set size), number of genes found with our analysis (Observed hits) and adjusted p-value. Universe size: proximal = 14,952; middle = 14,933; distal = 14,925. (PDF 258 kb) [file 12864_2016_2424_MOESM5_ESM.pdf]

Additional file 5

Section and responses	Gene set name	Gene set term	Set size	Observed hits	Adjusted p-value
Proximal (Linear)	GO:0006631	fatty acid metabolic process	68	19	<0.01
	GO:0006629	lipid metabolic process	184	33	<0.01
	GO:0006635	fatty acid beta-oxidation	21	9	<0.01
	GO:0042632	cholesterol homeostasis	33	11	<0.01
	GO:0030301	cholesterol transport	15	7	<0.01
	GO:0033344	cholesterol efflux	17	7	<0.01
	GO:0006695	cholesterol biosynthetic process	18	7	<0.01
	GO:0006637	acyl-CoA metabolic process	19	7	<0.01
	GO:0006694	steroid biosynthetic process	42	11	<0.01
	GO:0016126	sterol biosynthetic process	16	6	<0.01
	GO:0022900	electron transport chain	69	14	<0.01
	GO:0016192	vesicle-mediated transport	134	21	0.01
	GO:0010718	positive regulation of epithelial to mesenchymal transition	15	5	0.01
	GO:0015031	protein transport	386	45	0.01
	GO:0042157	lipoprotein metabolic process	15	5	0.01
	GO:0008203	cholesterol metabolic process	50	10	0.02
	GO:0034968	histone lysine methylation	22	6	0.02
	GO:0071300	cellular response to retinoic acid	17	5	0.02
	GO:0007040	lysosome organization	18	5	0.03
	GO:0055085	transmembrane transport	390	42	0.07
	GO:0006665	sphingolipid metabolic process	15	4	0.08
	GO:0006953	acute-phase response	22	5	0.09
Proximal (Logrithm)	GO:0006917	induction of apoptosis	131	16	0.06
Middle (Linear)	GO:0006631	fatty acid metabolic process	67	28	<0.01
	GO:0006629	lipid metabolic process	183	46	<0.01
	GO:0006635	fatty acid beta-oxidation	21	11	<0.01
	GO:0005975	carbohydrate metabolic process	141	32	<0.01
	GO:0022900	electron transport chain	69	18	<0.01
	GO:0015031	protein transport	387	60	<0.01
	GO:0042542	response to hydrogen peroxide	18	7	0.01
	GO:0009058	biosynthetic process	39	11	0.01
	GO:0043154	negative regulation of caspase activity	36	10	0.02
	GO:0055085	transmembrane transport	391	57	0.02
	GO:0050796	regulation of insulin secretion	21	7	0.02
	GO:0006006	glucose metabolic process	38	10	0.03
	GO:0007040	lysosome organization	18	6	0.04
	GO:0045471	response to ethanol	29	8	0.04
	GO:0006637	acyl-CoA metabolic process	19	6	0.04
	GO:0045444	fat cell differentiation	24	7	0.04
	GO:0016042	lipid catabolic process	69	14	0.05
	GO:0032091	negative regulation of protein binding	15	5	0.05
	GO:0043161	proteasomal ubiquitin-dependent protein catabolic process	32	8	0.07

	GO:0006520	cellular amino acid metabolic process	16	5	0.07
	GO:0034612	response to tumor necrosis factor	16	5	0.07
	GO:0001890	placenta development	22	6	0.08
	GO:0006953	acute-phase response	22	6	0.08
	GO:0051262	protein tetramerization	17	5	0.09
Middle (Logarithm)	GO:0071230	cellular response to amino acid stimulus	22	7	<0.01
	GO:0007160	cell-matrix adhesion	40	8	<0.01
	GO:0051259	protein oligomerization	22	6	<0.01
	GO:0007155	cell adhesion	341	25	<0.01
	GO:0006644	phospholipid metabolic process	17	4	0.01
	GO:0018149	peptide cross-linking	17	4	0.01
	GO:0045859	regulation of protein kinase activity	16	4	0.01
	GO:0034446	substrate adhesion-dependent cell spreading	18	4	0.01
	GO:0043065	positive regulation of apoptosis	165	14	0.01
	GO:0007229	integrin-mediated signaling pathway	55	7	0.02
	GO:0006749	glutathione metabolic process	24	4	0.04
	GO:0001937	negative regulation of endothelial cell proliferation	16	3	0.06
	GO:0030574	collagen catabolic process	16	3	0.06
	GO:0009411	response to UV	29	4	0.07
	GO:0043627	response to estrogen stimulus	29	4	0.07
	GO:0045766	positive regulation of angiogenesis	57	6	0.07
	GO:0030334	regulation of cell migration	30	4	0.08
	GO:0030168	platelet activation	19	3	0.09
	GO:0007179	transforming growth factor beta receptor signaling pathway	47	5	0.10
	GO:0042632	cholesterol homeostasis	33	4	0.10
	GO:0050731	positive regulation of peptidyl-tyrosine phosphorylation	47	5	0.10
	GO:0051897	positive regulation of protein kinase B signaling cascade	33	4	0.10
Middle (Exponential)	GO:0007243	intracellular protein kinase cascade	56	8	0.03
	GO:0030301	cholesterol transport	15	4	0.03
	GO:0006468	protein phosphorylation	426	27	0.08
	GO:0007049	cell cycle	411	26	0.08
	GO:0009615	response to virus	59	7	0.08
	GO:0031532	actin cytoskeleton reorganization	22	4	0.08
	GO:0006919	activation of caspase activity	50	6	0.09
	GO:0007067	mitosis	166	13	0.09
	GO:0008630	DNA damage response. signal transduction resulting in induction of apoptosis	15	3	0.09
Distal (Linear)	GO:0051260	protein homooligomerization	70	7	<0.01
	GO:0006953	acute-phase response	21	3	0.03
	GO:0016042	lipid catabolic process	69	5	0.03
	GO:0043085	positive regulation of catalytic activity	25	3	0.03
	GO:0006099	tricarboxylic acid cycle	16	2	0.05
	GO:0006520	cellular amino acid metabolic process	16	2	0.05
	GO:0006644	phospholipid metabolic process	17	2	0.05
	GO:0007200	activation of phospholipase C activity by G-protein coupled receptor protein signaling pathway coupled to IP3 second messenger	17	2	0.05

	GO:0008654	phospholipid biosynthetic process	37	3	0.05
	GO:0033344	cholesterol efflux	17	2	0.05
	GO:0042157	lipoprotein metabolic process	16	2	0.05
	GO:0045859	regulation of protein kinase activity	16	2	0.05
	GO:0051262	protein tetramerization	17	2	0.05
	GO:0051402	neuron apoptosis	16	2	0.05
	GO:0060271	cilium morphogenesis	16	2	0.05
	GO:0006869	lipid transport	43	3	0.06
	GO:0006958	complement activation. classical pathway	20	2	0.06
	GO:0008202	steroid metabolic process	41	3	0.06
	GO:0030307	positive regulation of cell growth	44	3	0.06
	GO:0006888	ER to Golgi vesicle-mediated transport	22	2	0.06
	GO:0009725	response to hormone stimulus	22	2	0.06
	GO:0006749	glutathione metabolic process	24	2	0.08
	GO:0016049	cell growth	24	2	0.08
	GO:0007224	smoothened signaling pathway	25	2	0.08
	GO:0008203	cholesterol metabolic process	50	3	0.08
	GO:0045785	positive regulation of cell adhesion	25	2	0.08
Distal (Exponential)	GO:0000910	cytokinesis	31	4	<0.01
	GO:0001824	blastocyst development	16	2	0.01
	GO:0043967	histone H4 acetylation	15	2	0.01
	GO:0019882	antigen processing and presentation	18	2	0.02
	GO:0030218	erythrocyte differentiation	24	2	0.03
	GO:0032313	regulation of Rab GTPase activity	27	2	0.03
	GO:0032851	positive regulation of Rab GTPase activity	28	2	0.03
	GO:0043154	negative regulation of caspase activity	36	2	0.06

Table A5: Over-represented Gene Ontology Biological Process (GOBP) terms associated with differentially expressed genes in the three intestinal sections. Genes are classified as linear, logarithmic or exponential, according to the best (i.e., with the smallest adjusted p-value) response type describing their expression pattern (as a function of fat intake). For each significant GOBP term (described by Gene set name and Gene set term) we summarize number of genes corresponding to the process (Set size), number of genes found with our analysis (Observed hits) and adjusted p-value. Universe size: proximal = 14,952; middle = 14,933; distal = 14,925.
