# Supplementary material for: Nonlinear transcriptomic response to dietary fat intake in the small intestine of C57BL/6J mice
Source: BMC Genomics. 2016 Feb 9;17:106. doi: 10.1186/s12864-016-2424-9 (PMC4748552; doi:10.1186/s12864-016-2424-9)
Supplement: Additional file 6: — Over-represented Gene Ontology Biological Process (GOBP) terms that can be found in various intestinal sections. For each GOBP term we specify up- and down-regulated genes (adjusted p-value < 0.1; however, most of the results of gene set enrichment analysis are well below this threshold - see Additional file 5). (PDF 296 kb) [file 12864_2016_2424_MOESM6_ESM.pdf]

Additional file 6

Sections and responses	GOBP	Proximal significant genes	Middle significant genes	Distal significant genes
Prox – Mid – Dist (all linear)	GO:0006953: acute-phase response	UP: *Saa3, Serpina1b*<br><br>DOWN: *Reg3b, Reg3g, Stat3*	UP: *Serpina1b*<br><br>DOWN: *Reg3b, Reg3g, Saa2, Stat3, Sigirr*	UP: *Serpina1b*<br><br>DOWN: *Saa1, Saa2*
Prox – Mid (all linear)	GO:0006629: lipid metabolic process	UP: *Acadl, Acadvl, Acads, Apoc2, Cpt2, Crat, Ldlr, Acer1, Acot2, Acot4, Acaa1b, Slc27a2, Acot1, Plcxd1, Ech1, Acaa2, Gde1, Hacl1, Pnpla8, Abhd5, Adipor2, Arv1, Crot, Acsl3, Echs1, Hadha*<br><br>DOWN: *Plcb3, Daglb, Scap, Npc1l1, Apob, Asah2, Serinc1*	UP: *Tecr, Hdlbp, Acadl, Acadvl, Acox1, Cpt1a, Cpt2, Crat, Fdxr, Gpx1, Hsd17b4, Lipa, Acer1, Acot4, Mttp, Pck1, Soat2, Acaa1b, Slc27a2, Slc27a4, Acot1, Hsd17b6, Acsl5, Ech1, Echdc2, Acaa2, Gde1, Hacl1, Lpin2, Lpin3, Pnpla8, Adipor2, Acot12, Gpcpd1, Acsl3, Acox2, Echs1, Hadha*<br><br>DOWN: *Pafah1b2, Ppard, Daglb, Gdpd5, Napepld, Asah2, Plb1, Plce1*	
	GO:0006631: fatty acid metabolic process	UP: *Acadl, Acadvl, Acads, Apoa2, Cd36, Cpt2, Crat, Prkar2b, Acaa1b, Slc27a2, Ech1, Acaa2, Pnpla8, Abhd5, Adipor2, Crot, Acsl3, Echs1, Hadha*<br><br>DOWN:	UP: *Acadl, Acadvl, Acox1, Cd36, Cpt1a, Cpt2, Crat, Faah, Fabp2, Hsd17b4, Lipa, Acaa1b, Slc27a2, Slc27a4, Mecr, Acsl5, Ech1, Echdc2, Acaa2, Lpin2, Lpin3, Pnpla8, Adipor2, Acot12, Acsl3, Acox2, Echs1, Hadha*<br><br>DOWN:	
	GO:0006635: fatty acid beta-oxidation	UP: *Acadvl, Eci1, Slc25a17, Eci2, Acaa2, Decr1, Bdh2, Echs1, Hadha*<br><br>DOWN:	UP: *Acadvl, Acox1, Hsd17b4, Pex5, Slc25a17, Eci2, Acaa2, Bdh2, Acox2, Echs1, Hadha*<br><br>DOWN:	
	GO:0006637: acyl-CoA metabolic process	UP: *Hmgcl, Acot8, Acot2, Acot4, Acot1, Oxsm*<br><br>DOWN: *Ces1d*	UP: *Hmgcl, Acot8, Acot4, Acot1, Oxsm, Acot12*<br><br>DOWN:	
	GO:0007040: lysosome organization	UP: *Cln8*<br><br>DOWN: *Abca1, Tpp1, Hexa, Cln6*	UP: *Acp2, Fam160a2*<br><br>DOWN: *Tpp1, Hexa, Hexb, Ppt1*	
	GO:0015031: protein transport	UP: *Kdelr3, Bet1, Nacad, Rab1, Rab6, Mcfd2, Sec22b, Sec23a, Timm17b, Vps45, Atg4d, Rabep1, Rab9, Vps29, Apba3, Cope, Xpo7, Sar1b, Gpr89, Yif1a, Golt1a, Snx4, Pex13, Senp2, Snx7*<br><br>DOWN: *Sft2d2, Gdi1, Cog1, Myo1c, Rab12, Vps33b, Rab8b, Scamp2, Gga3, Agap1, Copg2, Nup210, Sec61a2, Rab37, Rab32, Kdelr1, Ap2b1, Gga2, Chmp4b, Rrbp1*	UP: *Chmp7, Kdelr3, Sec13, Vps4a, Ap1m1, Arf4, Bet1, Pex5, Sec22b, Sec23a, Stam, Arcn1, Timm17a, Timm17b, Cox18, Bcap31, Eif5a, Serp1, Timm13, Ipo8, Uevld, Copg, Pex14, Copz1, Gosr2, Apba3, Rab2a, Cope, Tomm6, Sar1b, Kdelr2, Derl1, Yif1a, Golt1a, Sec62, Dopey2, Tmem48, Fam160a2, Senp2, Sil1*<br><br>DOWN: *Arrb1, Ap3d1, Gdi1, Mtm1, Ppt1, Rab17, Rab19, Rab33b, Rab3ip, Rufy1, Vps33b, Rab8b, Sec61a2, Rab37, Tomm40l, Duoxa2, Rab32, Rab27b, Rrbp1, Cog8*	
	GO:0022900: electron	UP: *Ndufb11, Etfb, Etfa,*	UP: *Etfb, Etfa, Fdxr,*	

	transport chain	Ndufa4, Ndufs8, Ndufs6, Cyb561d2, Ndufa7, Cyb5b, Uqcrfs1, Etfdh<br><br>DOWN: Cmah, Cyb561, Cyba	Ndufs8, Ndufs2, Ndufb6, Ndufs6, Cyb561d2, Ndufs5, Ndufa9, Cyb5b, Uqcrfs1, Etfdh, Ndufa5<br><br>DOWN: Cyb561, Frrs1, Enox2, Cyb561d1	
	GO:0055085: transmembrane transport	UP: Slc30a9, Aqp7, Aqp8, Cacna1d, Slc6a3, Sfxn1, Slc22a1, Slc23a1, Slc25a17, Slc6a2, Mfsd6l, Timm17b, Slc6a20b, Slc4a11, Gpr172b, Xpo7, Slc16a13, Abcb6, Senp2, Mfsd2a<br><br>DOWN: Abca1, Atp8a1, Slc7a1, Itpr3, Myo1c, Slc12a7, Slc19a1, Slc2a2, Mfsd4, Slc36a1, Slc7a9, Atp2a3, Slc4a4, Nup210, Abcb10, Sec61a2, Cacna1h, Slc30a7, Slc39a8, Slc29a3, Hvcn1, Rrbp1	UP: Slc25a45, Slc30a9, Slc35b1, Sec13, Aqp3, Aqp7, Abcc2, Slc6a3, Slc26a3, Sfxn1, Slc1a3, Slc22a1, Slc23a1, Slc25a17, Slc7a7, Mfsd7c, Timm17a, Timm17b, Catsper1, Slc6a20b, Slc16a12, Mfsd7a, Eif5a, Serp1, Timm13, Gpr172b, Pex14, Slc47a1, Sec62, Slc16a13, Slc39a11, Slc39a5, Tmem48, Abcb6, Abcb8, Senp2, Sil1<br>DOWN: Slc26a2, Kcnu1, Slc24a6, Slc1a1, Slc2a2, Slc5a1, Spns2, Trpm6, Slc5a9, Slc17a5, Slc7a9, Atp9b, Sec61a2, Tomm40l, Hiatl1, Mfsd1, Spns1, Hvcn1, Rrbp1, Slc4a10	
Prox – Mid (linear - logarithmic)	GO:0042632: cholesterol homeostasis	UP: Apoa2, Ldlr, Nr1h3, Pla2g10, Npc2<br><br>DOWN: Abca1, Npc1, Npc1l1, Apob, Abcg5, Abcg8	UP: Apoa4, Cav1, Pla2g10<br><br>DOWN: Cyp7a1	
Prox – Mid (linear - exponential)	GO:0030301: cholesterol transport	UP: Apoa2, Cd36, Ldlr, Npc2<br><br>DOWN: Abca1, Npc1l1, Apob	UP:<br><br>DOWN: Abca1, Abcg1, Scarb1, Stard3	
Mid – Dist (all linear)	GO:0006520: cellular amino acid metabolic process		UP: Cth, Acy1, Ddc, Got1<br><br>DOWN: Ccbl2	UP: Cth<br><br>DOWN: Tat
	GO:0016042: lipid catabolic process		UP: Lipa, Pla2g2c, Nceh1, Pnpla8, Ddhd2<br><br>DOWN: Ddhd1, Pafah1b2, Pla2g2a, Pld2, Ppt1, Daglb, Plb1, Plbd1, Plce1	UP: Apoc2<br><br>DOWN: Pla2g2a, Daglb, Pla2g2f, Plce1
	GO:0051262: protein tetramerization		UP: Me1, Pex5, Txnrd1<br><br>DOWN: Igf1r, Sbf2	UP: Me1<br><br>DOWN: Sbf2
Mid – Dist (linear - exponential)	GO:0043154: negative regulation of caspase activity		UP: Prdx3, Birc5, Gpx1, Igf1, Por<br><br>DOWN: Arrb1, Bcl2l1, Naip1, Igbp1, Usp47	UP: Birc5<br><br>DOWN: Igbp1
Mid – Dist (logarithmic - linear)	GO:0006644: phospholipid metabolic process		UP: Pla2g2d, Pla2g10, Pla2g12a, Ppap2b<br><br>DOWN:	UP:<br><br>DOWN: Pla2g2a, Pla2g2f
	GO:0006749: glutathione metabolic process		UP: Gpx3, Gstm1, Gstm3, Gstk1<br><br>DOWN:	UP: Cth, Hagh<br><br>DOWN:
	GO:0045859: regulation of protein kinase activity		UP: Prkar2b, Mtor<br><br>DOWN: Tsc1, Akap13	UP: Ect2<br><br>DOWN: Plce1

Table A6: Over-represented Gene Ontology Biological Process (GOBP) terms that can be found in various intestinal sections. For each GOBP term we specify up- and down-regulated genes (adjusted p-value < 0.1; however, most of the results of gene set enrichment analysis are well below this threshold - see Additional file 5).
