# Supplementary material for: Nonlinear transcriptomic response to dietary fat intake in the small intestine of C57BL/6J mice
Source: BMC Genomics. 2016 Feb 9;17:106. doi: 10.1186/s12864-016-2424-9 (PMC4748552; doi:10.1186/s12864-016-2424-9)
Supplement: Additional file 7: — Over-represented Gene Ontology Biological Process (GOBP) terms that are completely up-regulated. For each GOBP term we summarize: the total number of genes in the microarray (gene set size, labelled as Set size), the count of genes extracted with our analysis (observed hits, labelled as Hits), and the adjusted p-values. Intestine sections: Prox = proximal; Mid = middle; Dist = distal. Response types: lm = linear; log = logarithm; exp = exponential. (PDF 204 kb) [file 12864_2016_2424_MOESM7_ESM.pdf]

Additional file 7

Section and responses	GO ID	GOBP	Proximal			Middle			Distal		
			Set size	Hits	Adjusted p-value	Set size	Hits	Adjusted p-value	Set size	Hits	Adjusted p-value
Prox – Mid – Dist (all linear)	GO:0006749	glutathione metabolic process	24	5	0.01	24	6	0.01	24	2	0.04
	GO:0006888	ER to Golgi vesicle-mediated transport	22	4	0.05	22	5	0.02	22	2	0.03
Prox – Mid – Dist (lm – log -lm)	GO:0006749	glutathione metabolic process	24	5	0.01	24	4	<0.01	24	2	0.04
	GO:0042632	cholesterol homeostasis	33	5	0.06	33	3	0.04	33	2	0.08
Prox – Mid (lm - lm)	GO:0006629	lipid metabolic process	184	26	<0.01	183	38	<0.01			
	GO:0006631	fatty acid metabolic process	68	19	<0.01	67	28	<0.01			
	GO:0006635	fatty acid beta-oxidation	21	9	<0.01	21	11	<0.01			
	GO:0006637	acyl-CoA metabolic process	19	6	<0.01	19	6	<0.01			
	GO:0006695	cholesterol biosynthetic process	18	5	<0.01	18	4	0.04			
	GO:0008610	lipid biosynthetic process	81	10	0.01	81	11	0.02			
	GO:0016192	vesicle-mediated transport	134	13	0.03	134	19	<0.01			
	GO:0022900	electron transport chain	69	11	<0.01	69	14	<0.01			
	GO:0045454	cell redox homeostasis	47	6	0.07	47	9	<0.01			
	GO:0051289	protein homotetramerization	38	7	<0.01	38	6	0.07			
Prox – Mid (lm - log)	GO:0022900	electron transport chain	69	11	<0.01	69	5	0.03			
Prox – Mid (log - lm)	GO:0006631	fatty acid metabolic process	68	6	0.07	67	28	<0.01			
Mid – Dist (log - lm)	GO:0033344	cholesterol efflux				17	2	0.04	17	2	0.02
	GO:0042325	regulation of phosphorylation				34	3	0.04	34	2	0.08

Table A7: Over-represented Gene Ontology Biological Process (GOBP) terms that are completely up-regulated and can be found in various intestinal sections. For each GOBP term we summarize: the total number of genes in the microarray (gene set size, labelled as Set size), the count of genes extracted with our analysis (observed hits, labelled as Hits), and the adjusted p-values. Intestine sections: Prox = proximal; Mid = middle; Dist = distal. Response types: lm = linear; log = logarithm; exp = exponential.
