# Supplementary material for: Nonlinear transcriptomic response to dietary fat intake in the small intestine of C57BL/6J mice
Source: BMC Genomics. 2016 Feb 9;17:106. doi: 10.1186/s12864-016-2424-9 (PMC4748552; doi:10.1186/s12864-016-2424-9)
Supplement: Additional file 8: — Over-represented Gene Ontology Biological Process (GOBP) terms that include up-regulated genes only and can be found in various intestinal sections. Such GOBP terms can be found in various intestinal sections; lists of (up-regulated) differentially expressed genes associated with each GOBP term are summarized (adjusted p-value < 0.1; see Additional file 7). (PDF 286 kb) [file 12864_2016_2424_MOESM8_ESM.pdf]

Additional file 8

Section and responses	GOBP	Proximal significant genes	Middle significant genes	Distal significant genes
Prox – Mid – Dist (all linear)	GO:0006749: glutathione metabolic process	*Cth, Glo1, Hagh, Gstz1, Idh1*	*Cth, G6pdx, Ggt1, Hagh, Gpx1, Gstz1*	*Cth, Hagh*
	GO:0006888: ER to Golgi vesicle-mediated transport	*Bet1, Rab1, Sec22b, Sec23a*	*Sec13, Bet1, Sec22b, Sec23a, Lmf1*	*Sec22b, Lman1*
Prox – Mid – Dist (linear – logarithmic - linear)	GO:0006749: glutathione metabolic process	*Cth, Glo1, Hagh, Gstz1, Idh1*	*Gpx3, Gstm1, Gstm3, Gstk1*	*Cth, Hagh*
	GO:0042632: cholesterol homeostasis	*Apoa2, Ldlr, Nr1h3, Pla2g10, Npc2*	*Apoa4, Cav1, Pla2g10*	*Apoa4, Mttp*
Prox – Mid (all linear)	GO:0006629: lipid metabolic process	*Acadl, Acadvl, Acads, Apoc2, Cpt2, Crat, Ldlr, Acer1, Acot2, Acot4, Acaa1b, Slc27a2, Acot1, Plcxd1, Ech1, Acaa2, Gde1, Hacl1, Pnpla8, Abhd5, Adipor2, Arv1, Crot, Acsl3, Echs1, Hadha*	*Tecr, Hdlbp, Acadl, Acadvl, Acox1, Cpt1a, Cpt2, Crat, Fdxr, Gpx1, Hsd17b4, Lipa, Acer1, Acot4, Mttp, Pck1, Soat2, Acaa1b, Slc27a2, Slc27a4, Acot1, Hsd17b6, Acsl5, Ech1, Echdc2, Acaa2, Gde1, Hacl1, Lpin2, Lpin3, Pnpla8, Adipor2, Acot12, Gpcpd1, Acsl3, Acox2, Echs1, Hadha*	
	GO:0006631: fatty acid metabolic process	*Acadl, Acadvl, Acads, Apoa2, Cd36, Cpt2, Crat, Prkar2b, Acaa1b, Slc27a2, Ech1, Acaa2, Pnpla8, Abhd5, Adipor2, Crot, Acsl3, Echs1, Hadha*	*Acadl, Acadvl, Acox1, Cd36, Cpt1a, Cpt2, Crat, Faah, Fabp2, Hsd17b4, Lipa, Acaa1b, Slc27a2, Slc27a4, Mecr, Acsl5, Ech1, Echdc2, Acaa2, Lpin2, Lpin3, Pnpla8, Adipor2, Acot12, Acsl3, Acox2, Echs1, Hadha*	
	GO:0006635: fatty acid beta-oxidation	*Acadvl, Eci1, Slc25a17, Eci2, Acaa2, Decr1, Bdh2, Echs1, Hadha*	*Acadvl, Acox1, Hsd17b4, Pex5, Slc25a17, Eci2, Acaa2, Bdh2, Acox2, Echs1, Hadha*	
	GO:0006637: acyl-CoA metabolic process	*Hmgcl, Acot8, Acot2, Acot4, Acot1, Oxsm*	*Hmgcl, Acot8, Acot4, Acot1, Oxsm, Acot12*	
	GO:0006695: cholesterol biosynthetic process	*Ebp, Hmgcs2, Nsdhl, Pmvk, Tm7sf2*	*Ebp, G6pdx, Hmgcs2, Tm7sf2*	
	GO:0008610: lipid biosynthetic process	*Hsd17b11, Ebp, H2-Ke6, Hmgcs2, Hsd17b2, Nsdhl, Acss2, Pmvk, Oxsm, Tm7sf2*	*Tecr, Hsd17b11, Ebp, Hmgcs2, Mecr, Hsd17b12, Acss2, Ptplad2, Oxsm, Tm7sf2, Lass2*	
	GO:0016192: vesicle-mediated transport	*Kdelr3, Bet1, Rab1, Rab6, Mcfd2, Sec22b, Sec23a, Vps45, Cope, Sar1b, Ap4b1, Yif1a, Golt1a*	*Kdelr3, Sec13, Vps4a, Ap1m1, Arf4, Bet1, Sec22b, Sec23a, Arcn1, Bcap31, Copg, Copz1, Gosr2, Rab2a, Cope, Sar1b, Kdelr2, Yif1a, Golt1a*	
	GO:0022900: electron transport chain	*Ndufb11, Etfb, Etfa, Ndufa4, Ndufs8, Ndufs6, Cyb561d2, Ndufa7, Cyb5b, Uqcrfs1, Etfdh*	*Etfb, Etfa, Fdxr, Ndufs8, Ndufs2, Ndufb6, Ndufs6, Cyb561d2, Ndufs5, Ndufa9, Cyb5b, Uqcrfs1, Etfdh, Ndufa5*	
	GO:0045454: cell redox homeostasis	*1810046J19Rik, Ddit3, Txnrd2, 2810407C02Rik, Txndc15, Glrx5*	*1810046J19Rik, Gpx1, Txnrd1, 2810407C02Rik, Txndc15, Pdia6, Pdia5, Glrx5, Erp44*	
	GO:0051289: protein homotetramerization	*Acacb, Cth, Acadl, Acads, Aldoc, Dhps, Decr1*	*Cth, Acadl, Aldh1a1, Aldoc, Ide, Cda*	
Prox – Mid (linear - logarithmic)	GO:0022900: electron transport chain	*Ndufb11, Etfb, Etfa, Ndufa4, Ndufs8, Ndufs6, Cyb561d2, Ndufa7, Cyb5b, Uqcrfs1, Etfdh*	*Txn2, Ndufb9, Ndufc1, Ndufa7, Steap2*	

Prox- Mid (logarithmic - linear)	GO:0006631: fatty acid metabolic process	*Fabp2, Hsd17b4, Slc27a4, Acsl5, Acot12, Acox2*	*Acadl, Acadvl, Acox1, Cd36, Cpt1a, Cpt2, Crat, Faah, Fabp2, Hsd17b4, Lipa, Acaa1b, Slc27a2, Slc27a4, Mecr, Acsl5, Ech1, Echdc2, Acaa2, Lpin2, Lpin3, Pnpla8, Adipor2, Acot12, Acsl3, Acox2, Echs1, Hadha*	
Mid – Dist (logarithmic - linear)	GO:0033344: cholesterol efflux		*Apoa4, Cav1*	*Apoa4, Apoc2*
	GO:0042325: regulation of phosphorylation		*Pik3r3, Srpx2, Rptor*	*Pik3r3, Rptor*

Table A8: Over-represented Gene Ontology Biological Process (GOBP) terms that include up-regulated genes only. Such GOBP terms can be found in various intestinal sections; lists of (up-regulated) differentially expressed genes associated with each GOBP term are summarized (adjusted p-value < 0.1; see Additional file 7).
