# Supplementary material for: Nonlinear transcriptomic response to dietary fat intake in the small intestine of C57BL/6J mice
Source: BMC Genomics. 2016 Feb 9;17:106. doi: 10.1186/s12864-016-2424-9 (PMC4748552; doi:10.1186/s12864-016-2424-9)
Supplement: Additional file 9: — Over-represented Gene Ontology Biological Process (GOBP) terms that are completelydown-regulated. Data refer to GOBP terms that can be found in various intestinal sections. The following details are provided for each GOBP term: total number of genes in the microarray (gene set size, labelled as Set size), count of genes extracted with our analysis (observed hits, labelled as Hits), and adjusted p-values. Intestine sections: Prox = proximal; Mid = middle; Dist = distal. Response types: lm = linear; log = logarithm; exp = exponential. (PDF 204 kb) [file 12864_2016_2424_MOESM9_ESM.pdf]

Additional file 9

Section and responses	GO ID	GOBP	Proximal			Middle			Distal		
			Set size	Hits	Adjusted p-value	Set size	Hits	Adjusted p-value	Set size	Hits	Adjusted p-value
Prox – Mid – Dist (all linear)	GO:0045785	positive regulation of cell adhesion	26	4	0.05	26	5	0.08	25	2	0.03
Prox – Mid – Dist (lm – exp - lm)	GO:0003333	amino acid transmembrane transport	39	5	0.05	39	3	0.09	39	2	0.07
Prox – Mid (lm - lm)	GO:0007040	lysosome organization	18	4	0.02	18	4	0.08			
	GO:0009615	response to virus	59	7	0.03	59	9	0.07			
	GO:0030335	positive regulation of cell migration	83	10	0.01	83	10	0.08			
Prox – Mid (lm - exp)	GO:0007243	intracellular protein kinase cascade	56	7	0.03	56	6	0.01			
	GO:0009615	response to virus	59	7	0.03	59	5	0.04			
	GO:0030301	cholesterol transport	15	3	0.05	15	4	<0.01			
	GO:0033344	cholesterol efflux	17	4	0.01	17	3	0.03			
	GO:0042632	cholesterol homeostasis	33	6	0.01	33	3	0.07			
	GO:0046777	protein autophosphorylation	91	9	0.04	90	5	0.09			
	GO:0071300	cellular response to retinoic acid	17	5	<0.01	17	3	0.03			
Prox – Mid (log - lm)	GO:0009615	response to virus	59	6	0.04	59	9	0.07			
Prox – Mid (log - log)	GO:0043029	T cell homeostasis	15	3	0.03	15	2	0.08			
Prox – Mid (log - exp)	GO:0009615	response to virus	59	6	0.04	59	5	0.04			
	GO:0016358	dendrite development	18	4	0.01	20	2	0.09			
Mid - Dist (lm - lm)	GO:0006953	acute-phase response				22	5	0.07	21	2	0.02
	GO:0016042	lipid catabolic process				69	9	0.08	69	4	0.02

Table A9: Over-represented Gene Ontology Biological Process (GOBP) terms with down-regulated genes only. Data refer to GOBP terms that can be found in various intestinal sections. The following details are provided for each GOBP term: total number of genes in the microarray (gene set size, labelled as Set size), count of genes extracted with our analysis (observed hits, labelled as Hits), and adjusted p-values. Intestine sections: Prox = proximal; Mid = middle; Dist = distal. Response types: lm = linear; log = logarithm; exp = exponential.
