# Supplementary material for: Nonlinear transcriptomic response to dietary fat intake in the small intestine of C57BL/6J mice
Source: BMC Genomics. 2016 Feb 9;17:106. doi: 10.1186/s12864-016-2424-9 (PMC4748552; doi:10.1186/s12864-016-2424-9)
Supplement: Additional file 10: — Over-represented Gene Ontology Biological Process (GOBP) terms that include down-regulated genes only and can be found in various intestinal sections. For each term we specify the associated differentially expressed genes (adjusted p-value < 0.1; see Additional file 9). (PDF 281 kb) [file 12864_2016_2424_MOESM10_ESM.pdf]

Additional file 10

Section and responses	GOBP	Proximal significant genes	Middle significant genes	Distal significant genes
Prox – Mid – Dist (all linear)	GO:0045785: positive regulation of cell adhesion	*Fgf1, Cx3cl1, Tgm2, Vegfa*	*Fgf1, Itgav, Pld2, Cx3cl1, Tgm2*	*Saa1, Tgm2*
Prox – Mid – Dist (linear – exponential - linear)	GO:0003333: amino acid transmembrane transport	*Slc7a1, Slc1a5, Slc36a1, Slc7a9, Serinc1*	*Slco4a1, Slc3a2, Slc36a1*	*Slc1a1, Slc7a9*
Prox – Mid (all linear)	GO:0007040: lysosome organization	*Abca1, Tpp1, Hexa, Cln6*	*Tpp1, Hexa, Hexb, Ppt1*	
	GO:0009615: response to virus	*Pml, Mst1r, Mavs, Ddx58, Oas1b, Unc93b1, Ifih1*	*Bcl2l1, Ifit1, Ddx58, Rnasel, Trim56, Irf7, Zbp1, Ifih1, Zc3hav1*	
	GO:0030335: positive regulation of cell migration	*Acp5, Adam10, Adam17, Bmp2, Fgf1, Igf1r, Ptk2b, Cx3cl1, Vegfa, Zfp703*	*Adam10, Bmp2, Fgf1, Igf1r, Itgav, Pik3r1, Pld2, Ptk2b, Cx3cl1, Zfp703*	
Prox – Mid (linear - exponential)	GO:0007243: intracellular protein kinase cascade	*Socs3, Fgf1, Smpd2, Wnk1, Rps6ka4, Tnik, Dapk1*	*Socs3, Jak2, Mknk1, Slc9a1, Ick, Tnik*	
	GO:0009615: response to virus	*Pml, Mst1r, Mavs, Ddx58, Oas1b, Unc93b1, Ifih1*	*Mx1, Mx2, Pml, Mst1r, Mavs*	
	GO:0030301: cholesterol transport	*Abca1, Npc1l1, Apob*	*Abca1, Abcg1, Scarb1, Stard3*	
	GO:0033344: cholesterol efflux	*Abca1, Npc1, Abcg5, Abcg8*	*Abca1, Abcg1, Scarb1*	
	GO:0042632: cholesterol homeostasis	*Abca1, Npc1, Npc1l1, Apob, Abcg5, Abcg8*	*Abca1, Abcg1, Scarb1*	
	GO:0046777: protein autophosphorylation	*Clk2, Igf1r, Uhmk1, Lyn, Pak2, Wnk1, Map3k1, Taok3, Tnik*	*Jak2, Lyn, Pak1, Pim3, Tnik*	
	GO:0071300: cellular response to retinoic acid	*Abca1, Lyn, Ptk2b, Ptk6, Mll5*	*Abca1, Lyn, Rxrb*	
Prox –Mid (logarithmic - linear)	GO:0009615: response to virus	*Ifit1, Rnasel, Trim56, Irf7, Zbp1, Polr3f*	*Bcl2l1, Ifit1, Ddx58, Rnasel, Trim56, Irf7, Zbp1, Ifih1, Zc3hav1*	
Prox – Mid (all logarithmic)	GO:0043029: T cell homeostasis	*Bcl2l11, Fas, Stat5b*	*Bcl2l11, Gimap5*	
Prox – Mid (logarithmic - exponential)	GO:0009615: response to virus	*Ifit1, Rnasel, Trim56, Irf7, Zbp1, Polr3f*	*Mx1, Mx2, Pml, Mst1r, Mavs*	
	GO:0016358: dendrite development	*Mecp2, Pak1, Reln, Abi2*	*Pak1, Ss18l1*	
Mid – Dist (all linear)	GO:0006953: acute-phase response		*Reg3b, Reg3g, Saa2, Stat3, Sigirr*	*Saa1, Saa2*
	GO:0016042: lipid catabolic process		*Ddhd1, Pafah1b2, Pla2g2a, Pld2, Ppt1, Daglb, Plb1, Plbd1, Plce1*	*Pla2g2a, Daglb, Pla2g2f, Plce1*

Table A10: Over-represented down-regulated Gene Ontology Biological Process (GOBP) terms that can be found in various intestinal sections. For each term we specify the associated differentially expressed genes (adjusted p-value < 0.1; see Additional file 9).
